# Nationwide Age-Specific Changes in EMS-Transported Emergency Department Visits in Korea During the Pre-COVID-19 and Post-COVID-19 Periods

**DOI:** 10.3390/jcm15072552

**Published:** 2026-03-27

**Authors:** Min-Jung Kim, Jae-Hyun Kwon, Soo Hyun Park, Young-Hoon Byun, Ho-Young Song, Jin Hee Kim, Sung-Ha Kim, So-Hyun Paek

**Affiliations:** Department of Emergency Medicine, CHA Bundang Medical Center, CHA University, Seongnam 13496, Republic of Korea; mjtear8287@chamc.co.kr (M.-J.K.); suas11@chamc.co.kr (S.H.P.); byunyoun84@chamc.co.kr (Y.-H.B.); shyped85@chamc.co.kr (H.-Y.S.); heartfeltdoctor@chamc.co.kr (J.H.K.); paulie1018@chamc.co.kr (S.-H.K.)

**Keywords:** emergency medical services, ambulance transport, emergency department utilization, COVID-19, NEDIS, KTAS, pediatrics, post-arrival proxy indicators

## Abstract

**Background/Objectives**: The COVID-19 pandemic substantially changed emergency care utilization patterns, but nationwide evidence comparing age-specific changes in 119 EMS-transported emergency department (ED) visits between children and adults remains limited. Using nationwide data from Korea’s public EMS system, we evaluated pre-COVID-19 and post-COVID-19 changes in 119 EMS-transported ED visits using KTAS-defined emergency acuity, ED disposition, and ED length of stay (ED LOS). **Methods**: We conducted a nationwide retrospective observational study using the National Emergency Department Information System. We included all 119 EMS-transported ED visits from 1 January 2019 to 31 December 2020 and used 23 February 2020 as the index date. Children were aged <20 years and adults were aged ≥20 years. The primary outcome was KTAS-defined emergency acuity; secondary outcomes were ED disposition and ED LOS. Multivariable logistic regression analyses were performed separately by age group. **Results**: A total of 2,104,163 119 EMS-transported ED visits were included. The proportion of pediatric visits decreased from 9.3% to 6.8% after COVID-19. Among children, crude emergency acuity decreased, whereas hospital admission and ED mortality increased; after adjustment, the odds of emergency acuity and hospital admission were slightly higher in the post-COVID-19 period. Among adults, emergency acuity, hospital admission, ED mortality, and ED LOS all increased, and adjusted odds of emergency acuity and hospital admission were also higher. **Conclusions**: Children showed mixed changes across severity-related indicators, whereas adults demonstrated a more consistent post-COVID-19 shift toward a higher-acuity clinical profile. Because these indicators were measured after ED arrival, the findings should be interpreted cautiously. Further studies using linked prehospital and hospital data are needed to better evaluate age-specific changes in EMS use.

## 1. Introduction

Emergency medical services (EMS) are a core component of emergency care systems, providing timely transport of patients with acute illness or injury from the prehospital setting to the emergency department (ED). In Korea, prehospital rescue and transport are primarily provided through a single public system, the 119 ambulance service. Because public ambulance resources are finite and are intended primarily for time-sensitive conditions, whether 119 ambulance transport is aligned with patient need has long been an important policy and operational issue [1,2]. Prior Korean studies have suggested that both underuse (patients who would have benefited from ambulance transport but did not use it) and overuse (ambulance use despite relatively low clinical urgency) of public EMS occur, indicating that mismatch between patient need and ambulance utilization is not uncommon [1,2]. In pediatric patients, this issue may be even more complex because EMS use is influenced not only by the child’s clinical condition but also by caregiver perception, uncertainty, and access to alternative sources of care [3,4].

Coronavirus disease 2019 (COVID-19), first emerging in late 2019, was declared a pandemic by the World Health Organization (WHO) in March 2020 [5] and has led to marked changes in ED utilization and ambulance-transported ED visits worldwide. In Korea, both regional and nationwide studies have shown marked declines in ED visits after the onset of the pandemic, with particularly pronounced reductions in pediatric visits [6,7]. More recent nationwide data also showed that pediatric ED utilization in Korea remained below prepandemic levels for several years after the initial outbreak [8]. In addition, pandemic-related infection-control measures affected both prehospital and in-hospital processes. In Korea, patients with fever experienced prolonged EMS time intervals and markedly higher non-transport rates after the COVID-19 outbreak [9], while changes in ED-to-intensive care unit pathways and outcomes have also suggested substantial system-level disruption during the pandemic period [10].

However, direct assessment of prehospital transport necessity is difficult in large administrative datasets without detailed prehospital clinical information. Accordingly, studies using large-scale ED data may instead examine post-arrival indicators that indirectly reflect clinical urgency and downstream resource use. In this context, triage acuity, ED disposition, and ED length of stay (ED LOS) can be interpreted as indirect indicators, although not as definitive markers, of transport necessity and severity. This framing is particularly relevant in Korea, where a single nationwide EMS system and a unified ED surveillance database allow the assessment of population-level shifts in ambulance-transported ED utilization within a consistent healthcare system context.

Despite this background, nationwide evidence remains limited regarding age-specific changes in 119 EMS-transported ED visits across the pre-COVID-19 and post-COVID-19 periods in Korea. Therefore, we compared nationwide changes in 119 EMS-transported ED visits between children and adults across these two periods using KTAS category, ED disposition, and ED LOS as proxy indicators.

## 2. Materials and Methods

### 2.1. Research Design and Setting

This nationwide retrospective observational study used emergency department (ED) visit data from the National Emergency Department Information System (NEDIS), operated by the Ministry of Health and Welfare and the National Medical Center in Korea. NEDIS is a nationwide administrative database that collects standardized, real-time information on ED visits from regional and local emergency medical centers and emergency medical institutions across the country.

In Korea, prehospital emergency medical services (EMS) are primarily delivered through a single public system (119 EMS). Accordingly, this study analyzed ED visits transported by 119 EMS.

The study period was from 1 January 2019 to 31 December 2020. We prespecified 23 February 2020 as the index date because this was the date on which the Korean government raised the national infectious disease crisis alert to the highest level (“Serious”), marking a major nationwide escalation in the public health response to COVID-19 [11]. This date was used as an operational breakpoint to compare patterns of 119 EMS-transported ED visits before and after the nationwide intensification of the COVID-19 response. ED visits were categorized into a pre-COVID-19 period (1 January 2019 to 22 February 2020) and a post-COVID-19 period (23 February 2020 to 31 December 2020).

This study was approved for use of NEDIS data (NEDIS review No. 2022-05-01) and was reviewed by the Institutional Review Board of Bundang CHA Hospital (IRB No. 2022-04-063; approval date, 10 May 2022). The requirement for informed consent was waived because the study used de-identified secondary data.

### 2.2. Selection of Participants

Among all ED visits registered in NEDIS during the study period, we included visits with the arrival mode recorded as transport by 119 EMS. Visits arriving by private ambulance, walk-in, or other means were excluded. Each ED visit was treated as an independent event, even when the same patient had multiple visits.

Records with unknown reason for ED visit, missing age group information, unclassifiable or missing Korean Triage and Acuity Scale (KTAS) values, or unknown ED disposition were excluded from the final analytic cohort. In the NEDIS dataset, excluded KTAS records consisted of missing values (n = 65) and entries coded as “other” (n = 141), which could not be classified within the standard five-level KTAS framework.

For the primary stratified analyses, visits were classified as children (<20 years) and adults (≥20 years). This classification was based on the age-group structure available in the NEDIS dataset and was used as an operational definition for age-stratified analyses in the Korean clinical and administrative context. In addition, the predefined age categories available in NEDIS were used for age subgroup descriptions and for covariate adjustment in the multivariable models.

### 2.3. Data Collection and Measurements

For each 119 EMS-transported ED visit, we extracted demographic information (age and sex), visit date/time, visit reason (trauma vs. non-trauma), and insurance type (National Health Insurance, Medicaid type 1, Medicaid type 2, or other).

The initial Korean Triage and Acuity Scale (KTAS) level (1–5) at ED arrival was collected. KTAS is a five-level triage tool (level 1, resuscitation; level 2, emergent; level 3, urgent; level 4, less urgent; and level 5, non-urgent) used in Korean emergency departments [12]. For analysis, KTAS levels were dichotomized into emergency (levels 1–3) and non-emergency (levels 4–5) categories [12,13].

Chief complaints were categorized after examining their frequency distribution in the study population. Specifically, the 10 most frequent complaints were retained as separate categories, whereas all remaining complaints were combined into an “other” category to preserve interpretability and avoid excessive fragmentation of low-frequency categories. ED disposition was classified as discharge, transfer to another facility, admission to a general ward, admission to an intensive care unit, or death in the ED. ED length of stay (ED LOS) was calculated in minutes as the time from ED arrival to ED departure time (i.e., time of discharge, transfer, or admission decision).

Because direct assessment of prehospital transport necessity was not feasible from the dataset, the study evaluated post-arrival proxy indicators of patient acuity and downstream ED utilization. The primary outcome was the proportion of visits triaged as emergency (KTAS 1–3). The secondary outcomes were ED disposition categories and ED length of stay. These outcomes were interpreted as indirect indicators reflecting changes in case mix and clinical severity profile, and downstream ED utilization.

### 2.4. Data Analysis

All analyses were performed using SAS (version 9.4; SAS Institute Inc., Cary, NC, USA).

Continuous variables are presented as mean ± standard deviation, and categorical variables as frequency and percentage. Comparisons between the pre-COVID-19 and post-COVID-19 periods were conducted separately for children and adults. The chi-square test was used for categorical variables, and Student’s *t*-test for ED LOS. All tests were two-sided, and *p* < 0.05 was considered statistically significant.

To evaluate period-associated changes after adjusting for potential confounders, multivariable logistic regression analyses were performed separately for children and adults. The main adjusted outcomes were emergency triage (KTAS 1–3 vs. KTAS 4–5) and hospital admission (general ward or ICU admission vs. discharge).

Visits resulting in transfer to another facility or death in the ED were excluded from the admission model.

The primary independent variable was study period (post-COVID-19 vs. pre-COVID-19). Covariates included sex, age category, visit reason (trauma vs. non-trauma), and insurance type. In sensitivity analyses, we additionally adjusted the models for chief complaint category and, separately, for month of visit to partially account for temporal variation and seasonality.

### 2.5. Use of Generative Artificial Intelligence

Generative artificial intelligence (GenAI) tools (ChatGPT version 4o; OpenAI, San Francisco, CA, USA) were used only for language editing and improvement of manuscript readability during the preparation of this manuscript. The AI tools were not used for study design, data collection, data analysis, statistical modeling, interpretation of results, or generation of scientific conclusions. All analyses, interpretations, and final decisions regarding the content of the manuscript were performed and verified by the authors.

## 3. Results

### 3.1. Study Population

**Figure 1:**
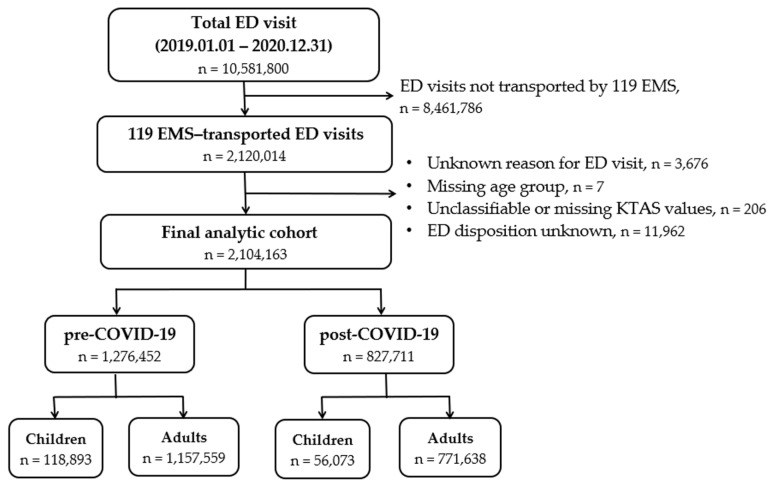
Flow diagram of selection of the final analytic cohort of 119 EMS-transported emergency department visits. ‘n’ indicates the number of visits.

Between 1 January 2019 and 31 December 2020, a total of 10,581,800 emergency department (ED) visits were recorded in the National Emergency Department Information System. Among these, 2,120,014 visits were transported by 119 emergency medical services (EMS). After applying the exclusion criteria shown in Figure 1, 2,104,163 visits were included in the final analytic cohort. Of these, 1,276,452 (60.7%) occurred during the pre-COVID-19 period and 827,711 (39.3%) during the post-COVID-19 period, as defined by the prespecified index date of 23 February 2020.

### 3.2. Age Distribution and Monthly Trends of 119-Transported ED Visits (Table 1 and Figure 2)

**Figure 2:**
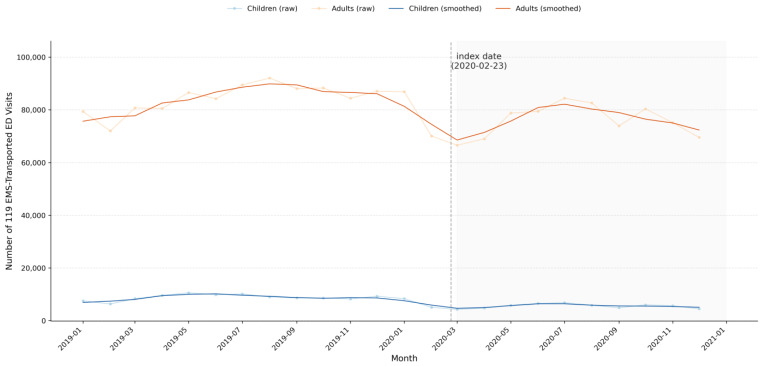
Monthly number of 119 EMS-transported emergency department visits in children and adults, January 2019 to December 2020. The vertical line indicates the prespecified index date of 23 February 2020, when the national infectious disease crisis alert in Korea was raised to the highest level (“Serious”). The light background represents the pre-COVID-19 period, and the dark background represents the post-COVID-19 period. Thin lines represent monthly counts and thick lines represent 3-month moving averages. Thin lines indicate monthly counts and thick lines indicate smoothed trends (3-month moving averages).

Table 1 summarizes changes in the age distribution of 119 EMS-transported ED visits in the pre-COVID-19 and post-COVID-19 periods, defined by the prespecified index date of 23 February 2020. The proportion of pediatric visits (<20 years) decreased from 9.3% in the pre-COVID-19 period to 6.8% in the post-COVID-19 period, whereas the proportion of adult visits (≥20 years) increased from 90.7% to 93.2%. Within the pediatric group, the proportion of children aged 1–4 years decreased from 33.0% to 25.4%, while the proportion of adolescents aged 10–19 years increased from 45.3% to 52.3%. Within the adult group, the proportion of older adults aged ≥65 years increased modestly from 43.2% to 44.5%. These age-specific shifts were consistent with the monthly trends shown in Figure 2. Following the prespecified index date of 23 February 2020, monthly 119 EMS-transported ED visits decreased sharply in both children and adults, with the most marked decline observed in March 2020, and then showed partial recovery over subsequent months.

### 3.3. Total ED Visits and the Proportion of 119 EMS-Transported Visits by Age Group (Figure 3)

**Figure 3:**
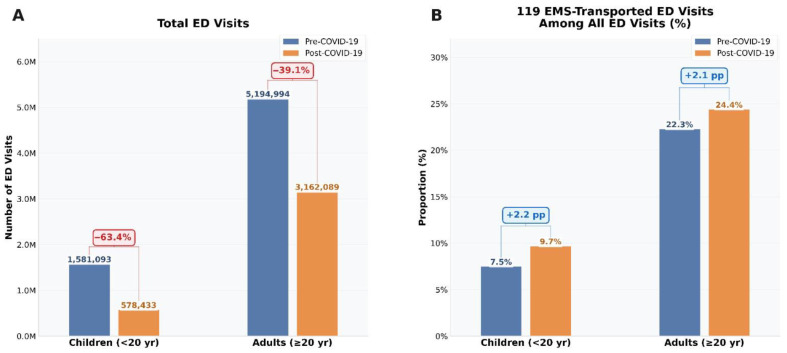
Total emergency department visits and the proportion of 119-transported ED visits in the pre- and post-COVID-19 periods among children and adults. (**A**) Total number of ED visits in the pre-COVID-19 and post-COVID-19 periods by age group. (**B**) Proportion of 119 EMS-transported ED visits among all ED visits in the pre-COVID-19 and post-COVID-19 periods by age group. The proportion was calculated as the number of 119 EMS-transported ED visits divided by the total number of ED visits within each age group. The percentage changes shown in panel A indicate the relative change in total ED visits between periods. The values shown in panel B indicate absolute changes in percentage points (pp) in the proportion of 119 EMS-transported ED visits between periods. Red labels indicate relative decreases, and blue labels indicate increases (percentage point changes in panel B).

Figure 3 compares total ED visits and the proportion of visits transported by 119 EMS among all ED visits in children and adults in the pre-COVID-19 and post-COVID-19 periods. In the post-COVID-19 period, the absolute number of total ED visits declined in both children and adults. In contrast, the proportion of 119 EMS-transported visits among all ED visits increased in both age groups, from 7.5% to 9.7% in children and from 22.3% to 24.4% in adults.

### 3.4. Clinical Characteristics and Chief Complaints Distribution Among 119 EMS-Transported ED Visits (Table 2)

Table 2 presents clinical characteristics and chief complaint distributions of 119 EMS-transported ED visits in children and adults in the pre-COVID-19 and post-COVID-19 periods. Among children, the proportion of trauma-related visits increased from43.8% to 52.7% in the post-COVID-19 period. Among the most frequent chief complaints, the proportions of seizure and fever decreased from 10.4% to 7.8% and from 9.7% to 6.7%, respectively. Among adults, the proportion of non-trauma visits increased slightly from65.7% to 66.5%. Among the most frequent chief complaints, fever increased from 3.5% to 4.5%, whereas dizziness decreased from7.5% to 7.2% (Table 2).

### 3.5. KTAS-Defined Emergency Acuity, ED Disposition, and Emergency Department Length of Stay (Table 3)

Table 3 summarize KTAS-definded emergency acuity at ED arrival, ED disposition, and ED length of stay (ED LOS) in children and adults during the pre-COVID-19 and post-COVID-19 periods. Among children, the proportion triaged as emergency (KTAS 1–3) decreased from 50.7% to 47.9%, whereas the proportion triaged as non-emergency (KTAS 4–5) increased from 49.3% to 52.1%. Among adults, the proportion triaged as emergency (KTAS 1–3) increased from 60.4% to 64.0%, while the proportion triaged as non-emergency (KTAS 4–5) decreased from 39.6% to 36.0%. In both age groups, the overall admission rate, defined as the sum of general ward and intensive care unit admissions, increased in the post-COVID-19 period (children: 16.0% to 17.4%; adults: 32.0% to 35.7%). ED mortality also increased in both children (0.4% to 0.6%) and adults (1.9% to 2.3%). ED LOS changed minimally in children (172.4 to 174.4 min) but increased in adults (265.9 to 293.4 min) (Table 3).

### 3.6. Adjusted Associations Between the COVID-19 Period and the Two Main Outcomes (Table 4 and Table 5)

Multivariable logistic regression analyses were conducted to evaluate the association between the COVID-19 period and the two main outcomes after adjustment for sex, visit reason (disease vs. non-disease), insurance type, and age category (Table 4 and Table 5). Chief complaint category was not included in the main models because it was considered more likely to reflect period-related case-mix changes than a baseline confounder. Additional sensitivity analyses adjusting for chief complaint category and, separately, for month of visit showed results materially similar to those of the main models (Appendix A, Table A1, Table A2, Table A3 and Table A4).

For emergency acuity, defined as KTAS 1–3, the adjusted odds were slightly higher during the post-COVID-19 period among children (adjusted OR 1.054, 95% CI 1.031–1.078) and increased more substantially among adults (adjusted OR 1.170, 95% CI 1.162–1.177) (Table 4).

Similarly, the adjusted odds of hospital admission were higher during the post-COVID-19 period in both children (adjusted OR 1.159, 95% CI 1.128–1.191) and adults (adjusted OR 1.191, 95% CI 1.184–1.199) (Table 5).

The interaction between COVID-19 period and age group was statistically significant for both outcomes (*p* < 0.0001).

## 4. Discussion

This nationwide study used NEDIS data to compare patterns of 119 EMS-transported emergency department (ED) visits between pediatric and adult populations during the pre-COVID-19 and post-COVID-19 periods in Korea, using KTAS-defined emergency acuity at ED arrival, ED disposition, and ED length of stay (ED LOS) as indirect proxy indicators related to transport appropriateness. Several previous NEDIS-based studies in Korea have examined the appropriateness or patterns of prehospital EMS use [1,2]. However, nationwide evidence directly comparing pediatric and adult EMS-transported ED visits within the same analytic framework across the pre-COVID-19 and post-COVID-19 periods has been limited. This broader comparative framework allowed us to identify age-specific differences in how these proxy indicators changed. In children, the findings were mixed: KTAS-defined emergency acuity did not change uniformly, whereas hospital admission and ED mortality increased during the post-COVID-19 period. In contrast, adults showed a more consistent pattern, with increases in KTAS-defined emergency acuity, hospital admission, ED mortality, and ED LOS, indicating a shift toward a higher-acuity clinical profile during the post-COVID-19 period. In children, the findings should be interpreted cautiously because the proxy indicators did not move uniformly. In the crude comparison, the proportion of visits classified as emergency acuity decreased, whereas hospital admission and ED mortality increased modestly. However, after adjustment for potential confounders, the odds of emergency acuity were slightly higher in the post-COVID-19 period. This divergence between crude and adjusted estimates likely reflects changes in case mix, particularly shifts in visit reason and the overall composition of pediatric EMS-transported visits. This interpretation is consistent with recent pediatric emergency care literature showing that, after the onset of COVID-19, pediatric ED utilization declined markedly, whereas changes in ambulance use, triage severity, and disposition did not necessarily move in parallel. In a recent nationwide Korean NEDIS analysis, overall pediatric ED utilization decreased substantially after the start of the pandemic, whereas admission and transfer rates remained relatively stable and in-hospital mortality increased [9]. Similarly, a 2024 multicenter study from Taiwan reported a marked reduction in pediatric ED visits and increased ambulance use during the pandemic, but no corresponding increase in the highest-acuity triage categories [14]. Together, these findings suggest that pediatric EMS-transported visits during the post-COVID-19 period reflected a selective and changing case mix rather than a simple, uniform increase or decrease in acuity. Previous Korean studies have also suggested that pediatric EMS use is strongly influenced by caregiver decision-making [3], and recent evidence indicates that ambulance use for low-acuity pediatric problems is shaped by access to care, perceived risk, and caregiver judgment [4]. These factors may have interacted with pandemic-related changes in healthcare-seeking behavior, thereby reshaping the composition of pediatric patients transported by 119 EMS. Among adults, the post-COVID-19 pattern was more consistent across the proxy indicators than that observed in children. In both crude and adjusted analyses, adult visits during the post-COVID-19 period were more likely to be classified as emergency acuity, and this pattern was accompanied by increases in general ward admission, intensive care unit admission, ED mortality, and ED length of stay. Taken together, these findings are consistent with a shift toward a higher-acuity clinical profile and greater downstream resource needs among adults transported by 119 EMS during the post-COVID-19 period. This interpretation is broadly supported by prior Korean studies reporting increased case severity and prolonged ED stay during the pandemic [15], prolonged prehospital time intervals among patients with fever [8], and disruption of ED-to-ICU pathways under pandemic conditions [10]. Recent studies from Korea and Japan have also shown worsening mortality-related outcomes among high-risk adult ED populations and ambulance-transported patients during the COVID-19 period [10,16]. Accordingly, the adult findings in the present study are best interpreted as reflecting a post-COVID-19 concentration of relatively sicker EMS users together with increased operational burden on the emergency care system.

Importantly, these findings should be interpreted in light of the fact that these proxy indicators were measured after ED arrival. KTAS-defined emergency acuity reflects triage status assigned in the emergency department and may not fully represent prehospital severity alone. Likewise, ED disposition and ED length of stay are influenced not only by clinical condition but also by hospital-level factors such as screening and isolation procedures, bed availability, admission thresholds, interfacility transfer delays, and overall ED crowding [10,15,17]. Accordingly, these variables should be regarded as complementary post-arrival proxy indicators of patient acuity and downstream ED utilization rather than direct measures of prehospital transport necessity [18,19]. This distinction is particularly important in the COVID-19 period, when operational strain may have affected both patient flow and disposition decisions. Therefore, our results are best interpreted as showing how the acuity and downstream course of 119 EMS-transported patients changed across the pre-COVID-19 and post-COVID-19 periods. This study has several limitations.

First, this was a retrospective observational study using NEDIS, and detailed prehospital clinical information, such as on-scene vital signs, symptom onset time, prehospital interventions, transport distance and time, and reasons for transport refusal or non-transport, was not available. Accordingly, it was not possible to directly determine prehospital transport necessity at the individual patient level. Therefore, KTAS-defined emergency acuity, ED disposition, and ED length of stay were used as indirect post-arrival proxy indicators and should not be interpreted as definitive measures of prehospital transport necessity.

Second, these proxy indicators were measured after ED arrival. KTAS is assigned in the emergency department and may not fully represent prehospital severity, whereas ED disposition and ED length of stay may be influenced not only by patient condition but also by hospital-level factors, including screening and isolation procedures, bed availability, institutional admission thresholds, interfacility transfer delays, and overall emergency department crowding during the COVID-19 period. These operational factors may have affected both triage-related and disposition-related outcomes.

Third, the analysis was conducted at the visit level rather than the patient level. As a result, repeated visits by the same individual could not be longitudinally linked, and changes in individual healthcare-seeking behavior or disease trajectory could not be assessed.

Fourth, the pre-COVID-19 and post-COVID-19 periods were unequal in duration, and potential seasonality or secular trends may not have been fully accounted for. To partially address this issue, we performed additional sensitivity analyses adjusting for month of visit, and the results were materially similar to those of the main models. Nevertheless, some of the observed differences between periods may still reflect temporal variation beyond the pandemic-related effect itself.

Fifth, although chief complaints were grouped according to frequency to improve interpretability in this large nationwide administrative dataset, this approach may have reduced clinical granularity. In particular, less frequent or non-specific presentations may have been absorbed into the “other” category, and residual confounding related to symptom composition may remain.

## 5. Conclusions

Using nationwide NEDIS data, we examined pre-COVID-19 and post-COVID-19 changes in 119 EMS-transported emergency department visits using KTAS-defined emergency acuity, ED disposition, and ED length of stay as indirect post-arrival proxy indicators. Children showed mixed changes across these indicators, whereas adults demonstrated a more consistent post-COVID-19 shift toward a higher-acuity clinical profile, with increases in emergency acuity, hospital admission, ED mortality, and ED length of stay. These findings should be interpreted cautiously because the study indicators were measured after ED arrival and do not directly establish prehospital transport necessity. Nevertheless, the results suggest that major public health disruptions may affect pediatric and adult EMS-utilizing populations differently. Further studies using linked prehospital and hospital clinical data are needed to better evaluate age-specific changes in EMS use patterns and post-arrival severity-related indicators.

## Figures and Tables

**Table 1:** Age distribution of nationwide 119 EMS-transported emergency department visits in the pre- and post-COVID-19 periods.

	Pre-COVID (N = 1,276,452)	Post-COVID (N = 827,711)	*p*-Value
Children, n (%)	118,893 (9.3)	56,073 (6.8)	
<1 year	8446 (7.1)	3777 (6.7)	<0.001
1–4 years	39,268 (33.0)	14,258 (25.4)	
5–9 years	17,371 (14.6)	8719 (15.6)	
10–19 years	53,808 (45.3)	29,319 (52.3)	
Adults, n (%)	1,157,559 (90.7)	771,638 (93.2)	
20–44 years	261,212 (22.6)	173,931 (22.5)	<0.001
45–54 years	172,671 (14.9)	109,644 (14.2)	
55–64 years	223,143 (19.3)	144,479 (18.7)	
≥65 years	500,533 (43.2)	343,584 (44.5)	

Values are presented as n (%). N indicates the total number of patients in each group, and n indicates the number of patients in each subgroup. Percentages for age subgroups were calculated within the pediatric and adult groups, respectively. Age subgroups were based on the predefined age-group categories in the NEDIS dataset. The index date was 23 February 2020. Pre-COVID-19 was defined as 1 January 2019 to 22 February 2020, and post-COVID-19 as 23 February 2020 to 31 December 2020. *p*-values were calculated using the chi-square test for age subgroup distributions within the pediatric and adult groups, respectively.

**Table 2:** Clinical characteristics and chief complaint distribution of nationwide 119 EMS-transported emergency department visits in the pre- and post-COVID-19 periods.

	Children			Adults		
	Pre-COVID (N = 118,893)	Post-COVID (N = 56,073)	*p*-Value	Pre-COVID (N = 1,157,559)	Post-COVID (N = 771,638)	*p*-Value
Gender, Male, n(%)	69,096 (58.1)	32,508 (58.0)	0.575	611,376 (52.8)	413,671 (53.6)	<0.001
Visit reason, n(%)						
Non-trauma	66,808 (56.2)	26,552 (47.4)	<0.001	760,828 (65.7)	512,984 (66.5)	<0.001
Trauma	52,085 (43.8)	29,521 (52.7)		396,731 (34.3)	258,654 (33.5)	
Insurance Type, n(%)						
National Health Insurance	100,067 (84.2)	47,205 (84.2)	<0.001	893,173 (77.2)	604,280 (78.3)	<0.001
Medicaid Type 1	2742 (2.3)	1428 (2.6)		120,581 (10.4)	81,836 (10.6)	
Medicaid Type 2	2163 (1.8)	1134 (2.0)		13,775 (1.2)	9562 (1.2)	
Others	13,921 (11.7)	6306 (11.3)		130,030 (11.2)	75,960 (9.8)	
Chief Complaints, n(%)			<0.001			<0.001
seizure	12,307 (10.4)	4392 (7.8)		13,600 (1.2)	9479 (1.2)	
Fever	11,577 (9.7)	3761 (6.7)		41,014 (3.5)	34,732 (4.5)	
abdominal pain	5780 (4.9)	2975 (5.3)		73,937 (6.4)	48,935 (6.3)	
headache	3577 (3.0)	1693 (3.0)		42,979 (3.7)	24,400 (3.2)	
syncope	3401 (2.9)	1631 (2.9)		30,475 (2.6)	16,493 (2.1)	
dyspnea	2567 (2.2)	1166 (2.1)		64,354 (5.6)	44,144 (5.7)	
dizziness	1585 (1.3)	791 (1.4)		86,648 (7.5)	55,227 (7.2)	
back pain	968 (0.8)	579 (1.0)		34,178 (3.0)	22,930 (3.0)	
chest pain	757 (0.6)	379 (0.7)		37,210 (3.2)	23,927 (3.1)	
general weakness	306 (0.3)	193 (0.3)		34,204 (3.0)	24,316 (3.2)	
Others	76,068 (64.0)	38,513 (68.7)		698,960 (60.4)	467,055 (60.5)	

Values are presented as n (%). N indicates the total number of patients in each group, and n indicates the number of patients in each subgroup. Percentages were calculated within each age group and study period. Chief complaints were categorized by selecting the 10 most frequent complaints in the dataset; all remaining complaints were grouped as “Other”. *p*-values were calculated using the chi-square test. Pre-COVID-19 was defined as 1 January 2019 to 22 February 2020, and post-COVID-19 as 23 February 2020 to 31 December 2020.

**Table 3:** KTAS-defined emergency acuity, ED disposition, and emergency department length of stay in children and adults across the pre- and post-COVID-19 periods.

	Children			Adults		
	Pre-COVID (N = 118,893)	Post-COVID (N = 56,073)	*p*-Value	Pre-COVID (N = 1,157,559)	Post-COVID (N = 771,638)	*p*-Value
Emergency acuity, n (%)						
Emergency (KTAS 1–3)	60,329 (50.7)	26,873 (47.9)	<0.001	699,267 (60.4)	493,980 (64.0)	<0.001
Non-emergency (KTAS 4–5)	58,564 (49.3)	29,200 (52.1)		458,292 (39.6)	277,658 (36.0)	
ED Disposition, n (%)						
Discharge	97,138 (81.7)	44,990 (80.2)	<0.001	720,392 (62.2)	450,977 (58.4)	<0.001
Transfer	2153 (1.8)	1000 (1.8)		45,033 (3.9)	26,851 (3.5)	
GW admission	17,170 (14.4)	8458 (15.1)		273,540 (23.6)	199,291 (25.8)	
ICU admission	1915 (1.6)	1281 (2.3)		96,832 (8.4)	76,601 (9.9)	
Death	517 (0.4)	344 (0.6)		21,762 (1.9)	17,918 (2.3)	
Length of Stay in ED (min, Mean ± SD)	172.4 ± 193.3	174.4 ± 202.4	<0.001	265.9 ± 452.0	293.4 ± 557.4	<0.001

Values are presented as n (%) unless otherwise indicated. N indicates the total number of patients in each group, and n indicates the number of patients in each subgroup. Percentages were calculated within each age group and study period. *p*-values for categorical variables were obtained using the chi-square test, and *p*-values for emergency department length of stay were obtained using Student’s *t*-test. Emergency department length of stay was analyzed using non-missing values only. Abbreviations: KTAS, Korean Triage and Acuity Scale; GW, general ward; ICU, intensive care unit; ED LOS, emergency department length of stay. Pre-COVID-19 was defined as 1 January 2019 to 22 February 2020, and post-COVID-19 as 23 February 2020 to 31 December 2020.

**Table 4:** Adjusted association between the COVID-19 period and emergency triage, stratified by age group.

Age Group	Comparison	Adjusted OR (95% CI)	*p*-Value
Children	Post-COVID-19 vs. Pre-COVID-19	1.054 (1.031–1.078)	<0.001
Adults	Post-COVID-19 vs. Pre-COVID-19	1.170 (1.162–1.177)	<0.001

KTAS-defined emergency acuity was defined as KTAS levels 1–3 and non-emergency acuity as KTAS levels 4–5. Multivariable logistic regression models were adjusted for sex, visit reason (non-trauma vs. trauma), insurance type, and age category. Chief complaint category was not included in the main model. Interaction between the COVID-19 period and age group: *p* < 0.0001.

**Table 5:** Adjusted association between the COVID-19 period and hospital admission, stratified by age group.

Age Group	Comparison	Adjusted OR (95% CI)	*p*-Value
Children	Post-COVID-19 vs. Pre-COVID-19	1.159 (1.128–1.191)	<0.001
Adults	Post-COVID-19 vs. Pre-COVID-19	1.191 (1.184–1.199)	<0.001

Hospital admission was defined as admission to a general ward or intensive care unit versus discharge. Visits resulting in transfer to another facility or death in the emergency department were excluded from this model. Multivariable logistic regression models were adjusted for sex, visit reason (non-trauma vs. trauma), insurance type, and age category. Chief complaint category was not included in the main model. Interaction between the COVID-19 period and age group: *p* < 0.0001.

## Data Availability

The datasets presented in this article are not readily available due to data access restrictions and privacy regulations. The authors are not permitted to share or redistribute the dataset. Requests to access the datasets should be directed to NEDIS approval procedures.

## Abbreviations

EMS	emergency medical services
NEDIS	National Emergency Department Information System
ED	emergency department
KTAS	Korean Triage and Acuity Scale
LOS	length of stay
ICU	intensive care unit
GW	general ward
COVID-19	coronavirus disease 2019
WHO	World Health Organization

## Appendix A. Sensitivity Analyses and Additional Methodological Notes

To assess the robustness of the main findings, we performed sensitivity analyses, additionally adjusting for chief complaint category and, separately, for month of visit. We also provide a brief supplementary note regarding the corrected KTAS categorization and excluded KTAS records.

**Table A1:** Adjusted association between the COVID-19 period and emergency acuity after additional adjustment for chief complaint category, stratified by age group.

Age Group	Comparison	Adjusted OR (95% CI)	*p*-Value
Children	Post-COVID-19 vs. Pre-COVID-19	1.071 (1.047–1.095)	<0.001
Adults	Post-COVID-19 vs. Pre-COVID-19	1.178 (1.170–1.186)	<0.001

Emergency acuity was defined as KTAS levels 1–3 and non-emergency acuity as KTAS levels 4–5..Models were adjusted for sex, visit reason, insurance type, age category, and chief complaint category.

**Table A2:** Adjusted association between the COVID-19 period and hospital admission after additional adjustment for chief complaint category, stratified by age group.

Age Group	Comparison	Adjusted OR (95% CI)	*p*-Value
Children	Post-COVID-19 vs. Pre-COVID-19	1.169 (1.137–1.201)	<0.001
Adults	Post-COVID-19 vs. Pre-COVID-19	1.178 (1.170–1.186)	<0.001

Hospital admission was defined as admission to a general ward or intensive care unit versus discharge. Visits resulting in transfer to another facility or death in the emergency department were excluded from this model. Models were adjusted for sex, visit reason, insurance type, age category, and chief complaint category.

**Table A3:** Adjusted association between the COVID-19 period and emergency acuity after additional adjustment for month of visit, stratified by age group.

Age Group	Comparison	Adjusted OR (95% CI)	*p*-Value
Children	Post-COVID-19 vs. Pre-COVID-19	1.032 (1.008–1.056)	<0.001
Adults	Post-COVID-19 vs. Pre-COVID-19	1.164 (1.156–1.172)	<0.001

Emergency acuity was defined as KTAS levels 1–3 and non-emergency acuity as KTAS levels 4–5. Models were adjusted for sex, visit reason, insurance type, age category, and month of visit.

**Table A4:** Adjusted association between the COVID-19 period and hospital admission after additional adjustment for month of visit, stratified by age group.

Age Group	Comparison	Adjusted OR (95% CI)	*p*-Value
Children	Post-COVID-19 vs. Pre-COVID-19	1.155 (1.122–1.188)	<0.001
Adults	Post-COVID-19 vs. Pre-COVID-19	1.197 (1.189–1.205)	<0.001

Hospital admission was defined as admission to a general ward or intensive care unit versus discharge. Visits resulting in transfer to another facility or death in the emergency department were excluded from this model. Models were adjusted for sex, visit reason, insurance type, age category, and month of visit.
